# Development and evaluation of a new *Plasmodium falciparum* 3D7 blood stage malaria cell bank for use in malaria volunteer infection studies

**DOI:** 10.1186/s12936-021-03627-z

**Published:** 2021-02-16

**Authors:** Stephen D. Woolley, Melissa Fernandez, Maria Rebelo, Stacey A. Llewellyn, Louise Marquart, Fiona H. Amante, Helen E. Jennings, Rebecca Webster, Katharine Trenholme, Stephan Chalon, Joerg J. Moehrle, James S. McCarthy, Bridget E. Barber

**Affiliations:** 1 QIMR Berghofer Medical Research Institute, Brisbane, QLD Australia; 2 Centre for Defence Pathology, Royal Centre for Defence Medicine, Joint Hospital Group, ICT Building, Birmingham Research Park, Vincent Drive, Birmingham, UK; 3 Clinical Sciences Department, Liverpool School of Tropical Medicine, Pembroke Place, Liverpool, UK; 4 School of Medicine, University of Queensland, Herston, QLD Australia; 5 Medicines for Malaria Venture, 20 Route de Pre-Bois, PO Box 1826, 1215 Geneva 15, Switzerland

**Keywords:** *Plasmodium falciparum*, Induced blood-stage malaria, CHMI, VIS, Malaria

## Abstract

**Background:**

New anti-malarial therapeutics are required to counter the threat of increasing drug resistance. Malaria volunteer infection studies (VIS), particularly the induced blood stage malaria (IBSM) model, play a key role in accelerating anti-malarial drug development. Supply of the reference 3D7-V2 *Plasmodium falciparum* malaria cell bank (MCB) is limited. This study aimed to develop a new MCB, and compare the safety and infectivity of this MCB with the existing 3D7-V2 MCB, in a VIS. A second bank (3D7-V1) developed in 1995 was also evaluated.

**Methods:**

The 3D7-V2 MCB was expanded in vitro using a bioreactor to produce a new MCB designated 3D7-MBE-008. This bank and 3D7-V1 were then evaluated using the IBSM model, where healthy participants were intravenously inoculated with blood-stage parasites. Participants were treated with artemether-lumefantrine when parasitaemia or clinical thresholds were reached. Safety, infectivity and parasite growth and clearance were evaluated.

**Results:**

The in vitro expansion of 3D7-V2 produced 200 vials of the 3D7-MBE-008 MCB, with a parasitaemia of 4.3%. This compares to 0.1% in the existing 3D7-V2 MCB, and < 0.01% in the 3D7-V1 MCB. All four participants (two per MCB) developed detectable *P. falciparum* infection after inoculation with approximately 2800 parasites. For the 3D7-MBE-008 MCB, the parasite multiplication rate of 48 h (PMR_48_) using non-linear mixed effects modelling was 34.6 (95% CI 18.5–64.6), similar to the parental 3D7-V2 line; parasitaemia in both participants exceeded 10,000/mL by day 8. Growth of the 3D7-V1 was slower (PMR_48_ of 11.5 [95% CI 8.5–15.6]), with parasitaemia exceeding 10,000 parasites/mL on days 10 and 8.5. Rapid parasite clearance followed artemether-lumefantrine treatment in all four participants, with clearance half-lives of 4.01 and 4.06 (weighted mean 4.04 [95% CI 3.61–4.57]) hours for 3D7-MBE-008 and 4.11 and 4.52 (weighted mean 4.31 [95% CI 4.16–4.47]) hours for 3D7-V1. A total of 59 adverse events occurred; most were of mild severity with three being severe in the 3D7-MBE-008 study.

**Conclusion:**

The safety, growth and clearance profiles of the expanded 3D7-MBE-008 MCB closely resemble that of its parent, indicating its suitability for future studies. *Trial Registration:* Australian New Zealand Clinical Trials registry numbers: P3487 (3D7-V1): ACTRN12619001085167. P3491 (3D7-MBE-008): ACTRN12619001079134

## Background

Malaria continues to cause major morbidity and mortality worldwide, with current control measures being threatened by the spread of artemisinin-resistance in the Greater Mekong Subregion [1–3]. New anti-malarial drugs and vaccines are, therefore, urgently required. The current anti-malarial drug pipeline has been accelerated by the use of human volunteer infection studies (VIS) [4–8], particularly the induced blood-stage malaria model (IBSM) [5, 6]. In these studies, healthy, malaria-naïve participants are inoculated with *Plasmodium*-infected erythrocytes, enabling the assessment of the blood stage schizont activity of antimalarial drug candidates [5, 6, 9–11]. As of March 2020, 401 volunteers have been inoculated with the *Plasmodium falciparum* 3D7 clone, most at QIMR Berghofer in Brisbane, Australia (n = 335), but some at sites in the Netherlands and UK (n = 66) [6, 12–16].

The current QIMR Berghofer malaria cell bank (MCB) used to inoculate volunteers with *P. falciparum*, termed 3D7-V2, was produced in 1995 [17–19]. At that time, two volunteers were experimentally infected by mosquito bite with *Plasmodium falciparum* 3D7, and 500 mL of blood was collected from each volunteer six hours following the onset of fever [19]. Although two MCBs were produced (3D7-V1 and 3D7-V2), the higher parasitaemia in the 3D7-V2 bank (0.1% compared to < 0.01% of erythrocytes parasitized, respectively) has led to this bank being utilized in subsequent malaria VIS. The 3D7-V1 has been utilized only once, for re-inoculation into the original donor [19].

Stocks of the *P. falciparum* 3D7-V2 MCB are limited, therefore, further MCBs are required to ensure an ongoing supply of this valuable resource. The development of further banks can be undertaken by collection of samples from malaria-infected patients or experimentally infected volunteers [17]. An alternative approach is the in vitro manufacture of banks using a bioreactor, such as the Wave ™ 25 bioreactor system [17]. This method has been used previously to produce and test in vivo two cell banks, a genetically modified *P. falciparum* blood stage-cell bank [17], and an arteminisin-resistant *P. falciparum* cell bank [20]. This proved to be a cost-efficient method for the production of a MCB for use in IBSM studies [17, 21]. This method also allows for blood group selection of the MCB.

The development of a new MCB, 3D7-MBE-008 (MBE-008), using this biomanufacture process, and the clinical evaluation of this MCB is reported. Safety, infectivity and parasite growth and clearance of the 3D7-MBE-008 and the previous 3D7-V1 were compared to the existing data on the 3D7-V2 bank.

## Methods

### Development of 3D7-MBE-008 Master Cell Bank

The 3D7-MBE-008 MCB was manufactured in accordance with Good Manufacturing Practice standards [22] in 2015 using the previously described method [17]. In brief, a single vial of the 3D7-V2 MCB was thawed and expanded using the bioreactor. Erythrocytes used in the production of the MCB were from a single blood group O Rh (D) negative donor, provided by Lifeblood (formerly Australian Red Cross Blood Service). The donor was screened in accordance with TGA regulatory requirements for donation of blood for transfusion. Pooled, heat inactivated serum collected from donors by Key Biologics (Memphis, Tennessee, U.S.) used in the manufacturing process was also extensively screened*.* The final 3D7-MBE-008 culture was cryopreserved with Glycerolyte 57 in 1:2.2 ratio, and aliquoted to produce 200 1 mL cryovials, which were stored between −140 and −196 °C in secure, monitored vapour phase liquid nitrogen tanks at Q-Gen Cell Therapeutics, Brisbane, Australia.

### Laboratory testing of the Master Cell Banks

The percentage of parasitized erythrocytes and the percentage of ring-stage parasites were determined via thin film microscopy for 3D7-MBE-008 and thick film microscopy for 3D7-V1. Testing for microbial contamination was performed in line with the British Pharmacopoeia Appendix XVI E- microbial contamination of cellular products [23].

Parasite viability of 3D7-MBE-008 was determined using flow cytometry as previously described [20] at the time of manufacture and then in an ongoing stability and sterility program, with testing every 12 months. The viability of the parasites in the 3D7-V1 bank was determined at the time of manufacture by limiting dilution assay followed by PCR as previously described [19].

For confirmation of parasite identity, the DNA sequence of three widely used hypervariable genes (*P. falciparum* merozoite surface protein-1 [*Pf* MSP-1], *Pf* MSP-2 and *Pf* glutate-rich protein [*Pf* GLURP]) from 3D7-MBE-008 were compared to 3D7-V2. In vitro drug sensitivity testing to nine antimalarials was also undertaken as previously described [19].

### Inoculum preparation

The viability of ring stage parasites in the MBE-008 MCB was assessed by flow cytometry as previously described [20], to identify the dilution required to achieve an inoculum dose similar to 3D7-V2 MCB. To prepare the inoculum, one or more vials of the MCB were thawed, with the resulting red cell pellet washed and resuspended in 0.9% sodium chloride. The washed cell suspension was then diluted with 0.9% sodium chloride to achieve the target number of viable ring-stage parasites in each 2 ml inoculum, taking into account the characteristics of each MCB including the percentage of parasitized erythrocytes, the percentage of ring-stage erythrocytes, and parasite viability. The number of parasites in the final inoculum was verified by 18S quantitative PCR targeting the *P. falciparum* 18S rRNA gene (qPCR) [24] with results available after inoculation.

### Clinical study design

Two concurrent IBSM studies were conducted, one with the 3D7-V1 MCB and the other with 3D7-MBE-008. Each study consisted of two single-participant cohorts, with a 5-week period between cohorts. The primary objective of both studies was safety. Secondary objectives included infectivity, parasite growth and clearance, the latter following administration of artemether-lumefantrine.

The studies were conducted at Q-Pharm Pty Ltd, Brisbane, Australia. Ethical approval was given by QIMR Berghofer Human Research Ethics Committee (HREC), and by Lifeblood HREC for *P. falciparum* 3D7-MBE-008. All participants gave written informed consent before enrolment. Both studies were registered with the Australian New Zealand Clinical Trials registry; 3D7-V1 (ACTRN12619001085167) and 3D7-MBE-008 (ACTRN12619001079134).

### Participants

Participants were eligible if they were aged 18–55 years, healthy and malaria-naïve (see Additional file 1: Appendix for inclusion and exclusion criteria). For the 3D7-V1 study, only males who were blood group Rh(D) positive were eligible, due to the fact that the 3D7-V1 MCB donor was Rh(D) positive.

### Procedures

**Fig. 1:**
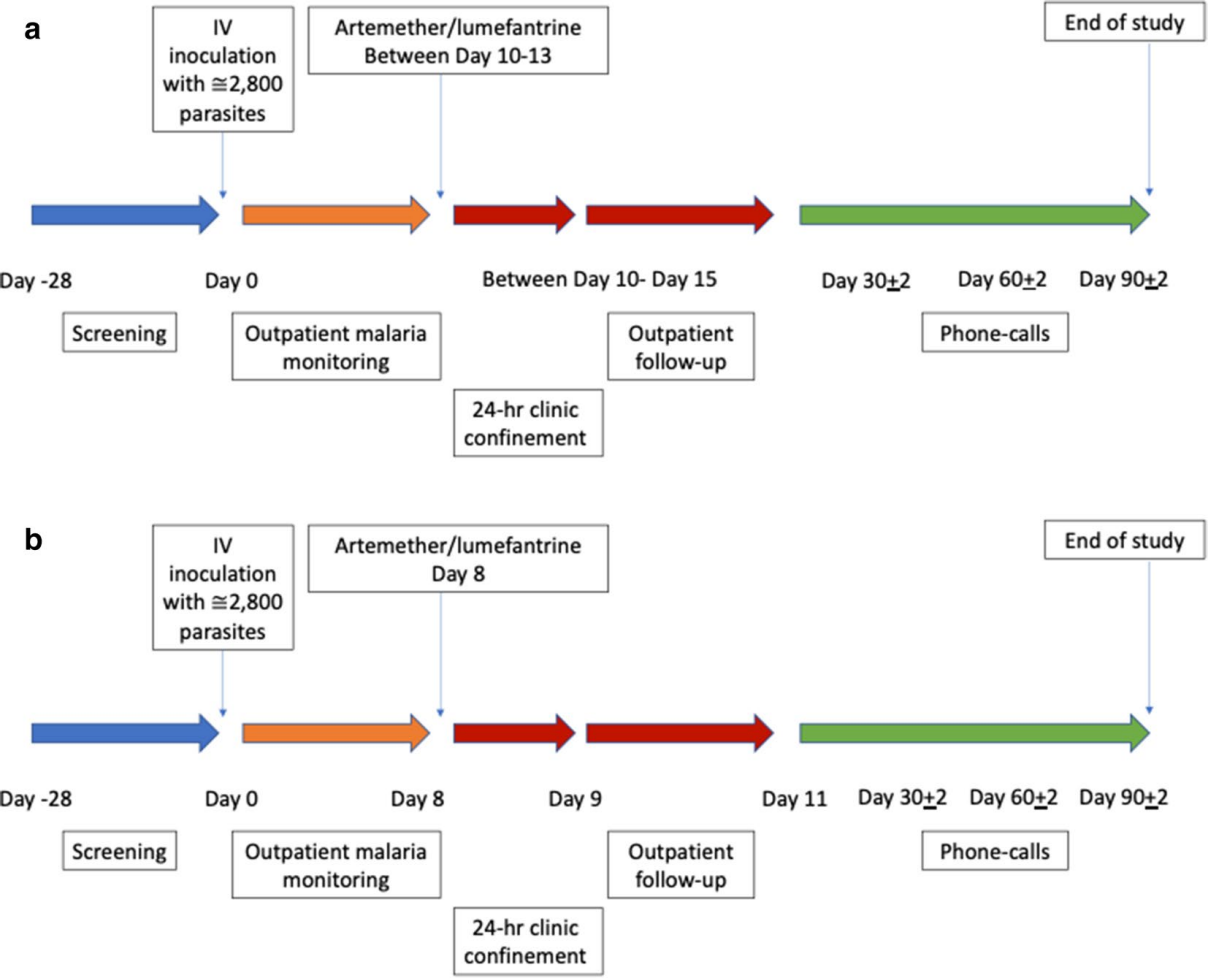
Study Design. In both studies, participants were inoculated with approximately 2800 infected erythrocytes on Day 0, followed by artemether-lumefantrine treatment when threshold parasitaemia was reached (> 10,000 parasites/mL). The participants were confined for 3 days, during which they were treated with artemether-lumefantrine. The last study visit occurred on Day 90 ± 2. For the 3D7-V1 study, the confinement period occurred between Day 10–13 (Panel **a**). For the 3D7-MBE-008 study, the confinement period commenced on Day 8 (Panel **b**)

All participants were inoculated with approximately 2800 viable infected erythrocytes on Day 0. Parasitaemia was monitored by 18S qPCR daily [24] from Day 4 until parasites were detected, then twice daily until artemether-lumefantrine (20 mg artemether/ 120 mg lumefantrine; Novartis Pharmaceuticals Pty Ltd) was given, and then at specified timepoints post treatment until qPCR was negative (Additional file 1: Table S1). Figure 1a illustrates the study design for 3D7-V1 MCB and Fig. 1b illustrates the study design for 3D7-MBE-008 MCB. The protocol specified that a curative course of artemether-lumefantrine, consisting of 6 doses of 4 tablets over a 60-h period, would be given when the participants’ parasitaemia exceeded 10,000 parasites/mL, or the participants’ malarial clinical score was ≥ 6 (see Additional file 1 for calculation of malaria clinical score). However, due to slower than expected parasite growth in the first subject inoculated with 3D7-V1, and lack of any clinical symptoms in the participant at a parasite count of 10,000 parasites/mL, the 3D7-V1 protocol was amended to change the parasite treatment threshold to 100,000 parasites/mL.

### Safety assessments

Safety was evaluated by recording all adverse events as well as any abnormal laboratory results. Investigations were performed at the timepoints specified in Additional file 1: Table S1. During every outpatient visit and during confinement, a malaria clinical score for each participant was generated. A graded assessment of symptoms and laboratory results was used (see Additional file 1).

### Parasite growth and clearance

The parasite multiplication rate of 48 h (PMR_48_) for each MCB was calculated by applying the pre-treatment qPCR data to a sine-wave growth model, estimated using a non-linear mixed effects model in R Statistical package 3.6.1, as previously reported [25]. The PMR_48_ for each MCB was then presented as an estimate with a 95% CI. To determine if there were any significant differences between the growth model parameter estimates from the new MCBs and the previously used 3D7-V2 MCB, an omnibus test for between-group differences was used [26]. The sine- wave growth model estimated using a non-linear regression model was also used to retrospectively calculate the parasite growth characteristics of 3D7-V1 in the initial volunteer re-infected with his isolate (3D7-V1) in 1995. The parasite clearance profiles post artemether-lumefantrine treatment for each participant were estimated from the slope of best fit of the parasite clearance rate and transformed to estimate the parasite reduction ratio (PRR) per 48 h in the logarithmic-scale (log_10_PRR_48_) and the parasite clearance half-life as previously reported [27], using R Statistical package 3.6.1. Parasite clearance parameters for each bank are summarized as a weighted mean and corresponding 95% CI estimated using the inverse variance method as detailed in [27].

## Results

### MCB characteristics

The blood used for the biomanufacture of 3D7-MBE-008 tested negative for microbial contamination and for serologic evidence of infective agents. Manufacture was completed in November 2015 and produced 200 vials. The analysis of the three genetic markers (*Pf msp*-1, *Pf msp*-2 and *Pf glurp*) showed that no changes had taken place between the starting 3D7-V2 MCB and resulting 3D7-MBE-008 MCB, ruling out high level genetic change during the biomanufacturing process. The in vitro drug sensitivity of the 3D7-MBE-008 MCB showed the same drug sensitivity profile as the established parental line to nine antimalarials (sensitive to amodiaquine, atovaquone, artemisinin, chloroquine, lumefantrine, piperaquine, pyronaridine and quinine; resistant to mefloquine). The parasite concentration of 3D7-MBE-008 MCB was 4.3%, with 96% of parasites in ring-stage.

Viability of the 3D7-MBE-008 MCB at 12, 24 and 36 months (prior to use) was 83%, 71% and 63%, respectively. Microbial contamination testing at these time points was negative. The parasitaemia of 3D7-V1 at the time of collection was 0.01%. The viability as measured by limit dilution and PCR [19] at the time of manufacture was approximately 34%.

### Study participants

The studies were conducted between August 2019 and December 2019.Two participants were enrolled into each study. A total of 22 potential volunteers were screened for the 3D7-MBE-008 study and 19 for 3D7-V1. All four inoculated participants completed the study and are included in the safety and parasite profile analysis. The participants for the 3D7-MBE-008 study were a 32-year-old white male and a 31-year-old white non-pregnant female. The participants for the 3D7-V1 study were both 19-year-old white males.

### Inoculation and parasite growth

**Fig. 2:**
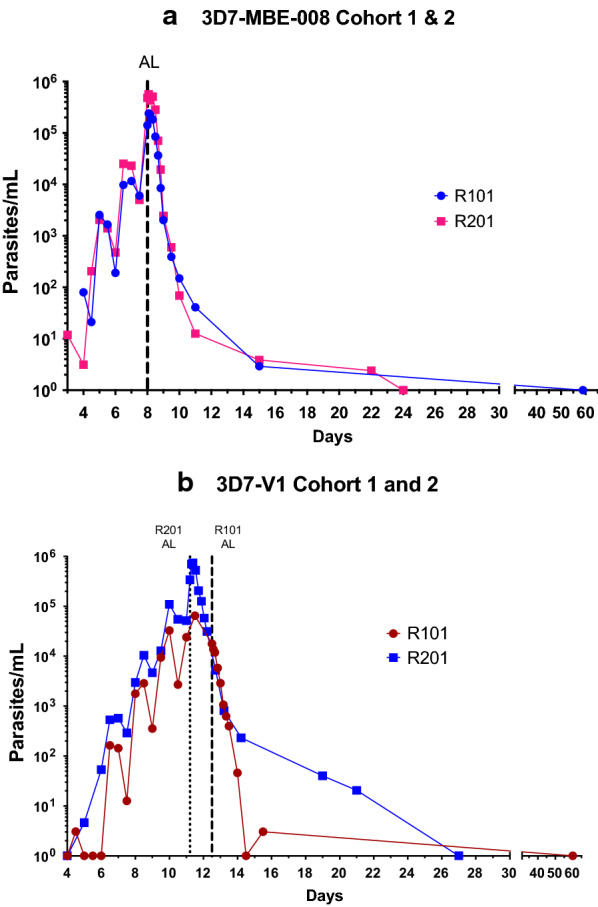
Parasite growth and clearance after artemether-lumefantrine (AL) treatment Parasitaemia from Day 4 to Day 60 for the 3D7-MBE-008 study (Panel **a**). Parasitaemia from Day 4 to Day 60 for the 3D7-V1 study (Panel **b**). The vertical dashed lines represent the administration of artemether-lumefantrine treatment

In the 3D7-MBE-008 study, the number of parasites in each of the inocula, determined retrospectively by 18S qPCR, was 18,700 and 23,100. Both participants had parasitaemia detectable by 18S qPCR on Day 4 and reached threshold parasitaemia (> 10,000 parasites/mL) on Day 8, when artemether-lumefantrine was commenced. The parasite counts prior to treatment were 141,416 parasites/mL and 480,871 parasites/mL, with peak parasitaemia reached for both subjects at 2 h post administration of artemether-lumefantrine (239,278 parasites/mL and 563,886 parasites/mL) (Fig. 2). Using the non-linear mixed effects model estimates, the estimated PMR_48_ was 34.6 (95% CI 18.5–64.6) (Table 1), similar to that reported in a large meta-analysis of the growth rate of 3D7-V2 in previous VIS, which is 31.9 (95% CI 28.7–35.4) [25]. There were no significant differences between the growth parameters of 3D7-MBE-008 (n = 2) and 3D7-V2 (n = 177) (see Additional file 1: Table S2).

**Table 1:** Summary of parasite growth and clearance characteristics

Parameter	3D7-V1 (n = 2) Estimate (95% CI)	3D7-MBE-008 (n = 2) Estimate (95% CI)	3D7-V1 1995 (n = 1) Estimate (95% CI)	3D7-V2 (n = 177) Estimate (95% CI) [25]
PMR_48_	11.5 (8.5–15.6)	34.6 (18.5–64.6)	6.4 (4.6–8.8)	31.9 (28.7–35.4)
Lifecycle (hours)	36.5 (35.0–38.0)	38.8 (36.7–40.8)	42.5 (39.2–45.8)	38.8 (38.3–39.2)
Growth rate (log_10_ parasites/day)	0.52 (0.30–0.74)	0.80 (0.54 –1.10)	0.35 (0.13–0.58)	0.63 (0.59–0.66)
PRR_48_	2254 (1705–2979)	3806 (1446–10018)	_	_
log10 PRR_48_	3.35 (3.23–3.47)	3.58 (3.16–4.00)	_	_
Parasite clearance half life	4.31 (4.16–4.47)	4.04 (3.61–4.57)	_	_

n, number of participants; CI, 95% confidence intervals; PMR, parasite multiplication rate; PRR, parasite reduction ratio. Growth parameter estimates are calculated from a non-linear mixed effects model. Clearance parameter estimates (PRR_48_, log_10_ PRR_48_ and parasite clearance half-life) are calculated from a weighted mean of individual clearance parameters estimates

In the 3D7-V1 study, the number of parasites in each inocula, determined retrospectively by qPCR, was approximately 3050 and 2694 respectively. Participant one developed detectable parasitaemia on day 5, whereas the second participant had detectable parasitaemia on day 4. The parasitaemia in both participants increased more slowly than those inoculated with 3D7-MBE-008, despite them being derived from the same parental 3D7 clone; on day 8, when treatment was expected to be required, the parasitaemias of the two participants were only 1776 parasites/mL and 2982 parasites/mL, respectively, with malaria clinical scores of zero in both participants. An urgent protocol amendment was approved by the HREC to allow treatment to be administered at a parasite threshold of > 100,000 parasites/mL, and the two participants were, therefore, treated on days 12 (first participant) and day 11 (second participant). For the first participant the pre-treatment parasite count was 17,699 parasites/mL, and the peak parasite count (day 11) was 64,786 parasites/mL. For the second participant, the pre-treatment parasite count was 340,789 parasites/mL, and the peak parasite count (occurring 4 h post artemether-lumefantrine) was 742,813 parasites/mL. The estimated PMR_48_ for 3D7-V1 was 11.5 (95% CI 8.5–15.6) (Table 1). The primary parasitaemia data from the original 3D7-V1 subject that had been calculated using a different method [19] was retrieved. Using these data in the sine wave growth model, the PMR_48_ for 3D7-V1 in the initial volunteer re-infected in 1995 was 6.4 (95% CI 4.6–8.8) (Table 1). Due to the heterogeneity in the method of calculation of parasitaemia between these studies, a combined analysis was not performed. The tests of heterogeneity of the individual growth parameters of the 3D7-V1 and existing 3D7-V2 MCB showed a significant difference between the parasite growth rate (p < 0.001) and parasite lifecycle (p ≤ 0.001) (Additional file 1: Table S3)**.**

### Parasite clearance

Parasitaemia cleared in all four participants following the administration of artemether-lumefantrine. In the 3D7-MBE-008 study, the parasite clearance half-lives in the two participants were 4.01 and 4.06 (weighted mean 4.04, 95% CI 3.61–4.57) hours, and the log_10_PRR_48_′s were 3.60 and 3.56 (weighted mean 3.58, 95% CI 3.16–4.00) h. In the 3D7-V1 study, the parasite clearance half-lives in the two participants were 4.11 and 4.52 (weighted mean 4.31, 95% CI 4.16–4.47) hours and the log_10_PRR_48_′s were 3.52 and 3.20 (weighted mean 3.35, 95% CI 3.23–3.47) h in the 3D7- V1 study (Table 1).

### Adverse events

There were 35 reported adverse events in the 3D7-MBE-008 study and 24 in the 3D7-V1 study (see Table 2). In the 3D7-MBE-008 study the majority of the adverse events were mild or moderate (32/35, 91.4%) and attributable to early malaria (25/35, 71.4%); three were graded as severe (two episodes of lymphopenia; see below) and one of raised alanine transaminase [ALT]; see below). In the 3D7-V1 study, the majority of adverse events were mild (14/24, 58.3%), with the remainder being moderate; nearly all were attributable to early malaria (23/24, 95.8%). There were no serious adverse events reported in either study. The most common adverse events reported across both studies were chills (n = 6), headaches (n = 6) and myalgia (n = 7). One of the participants in the 3D7-MBE-008 study had a maximum malaria clinical score of 9 (8 h post artemether/lumefantrine) (see Table S4). One of the participants in the 3D7-V1 study had a maximum score of 8 (36 h post artemether/lumefantrine) (see Additional file 1: Table S5).

**Table 2:** Adverse events reported during the studies

Adverse event	3D7-V1 (N = 2) n (M)	3D7-MBE-008 (N = 2) n (M)
Systemic		
Arthralgia	2 (2)	0 (0)
Chills	2 (3)	2 (3)
Decreased appetite	1 (1)	1 (1)
Fatigue	1 (2)	0 (0)
Feeling hot	1 (1)	1 (1)
Headache	2 (3)	2 (3)
Lethargy	0 (0)	1 (1)
Malaise	1 (1)	0 (0)
Myalgia	2 (3)	2 (4)
Pyrexia	2 (2)	2 (3)
Sweating	2 (2)	1 (3)
Tachycardia	1 (1)	1 (2)
Laboratory abnormalities		
ALT increased	1 (1)	1 (1)
AST increased	0 (0)	1 (1)
Lymphocyte count decreased	1 (1)	2 (1)
Neutrophil count decreased	0 (0)	1 (1)
Gastrointestinal		
Abdominal pain	0 (0)	2 (2)
Constipation	0 (0)	1 (1)
Diarrhoea	0 (0)	2 (2)
Other		
Back pain	0 (0)	1 (1)
Erythema (from tape)	0 (0)	1 (1)
Pain (venepuncture site)	0 (0)	1 (1)
Ulcer (lip)	0 (0)	1 (1)
Upper respiratory tract infection	1 (1)	0 (0)

N, total number of participants in each cohort; n, number of participants reporting the adverse event; M, number of occurrences of adverse events. Adverse events were coded to System Organ Class and Preferred Term using MedDRA Version 20.1

One participant in each study developed a raised ALT. One participant in the 3D7-MBE-008 study had a peak ALT on Day 11 of 191 U/L (4.8 × ULN [upper limit of normal]) which normalized by Day 59. The peak aspartate aminotransferase (AST), also on Day 11, was 122 U/L (3.1 × ULN) and it normalized on Day 15. The bilirubin was normal. In the 3D7-V1 study, one participant had a peak ALT on Day 14 of 128 U/L (3.2 × ULN) which normalized by Day 27. The AST and bilirubin were not significantly raised.

Three participants developed transient falls in white cell counts that were classified as moderate or severe: lymphopenia (3D7-V1, nadir of 0.42 × 10^9^/L, lower limit of normal [LLN] = 1.0 × 10^9^/L]; 3D7-MBE-008, nadir of 0.34 × 10^9^/L and 0.39 × 10^9^/L); neutropenia (3D7-MBE-008 n = 1, 1.35 × 10^9^/L, LLN = 1.5 × 10^9^/L). These transient reductions were attributed to malaria.

## Discussion

Here we report the manufacture and evaluation of a new *P. falciparum* MCB that can be utilized in future IBSM studies. In vivo testing of 3D7-MBE-008 MCB, and the previously manufactured 3D7-V1 MCB, indicated that they were well tolerated in healthy, malaria-naïve participants. The PMR_48_ for the 3D7-MBE-008 MCB was comparable to the existing 3D7-V2 MCB. In contrast, the 3D7-V1 MCB had a slower PMR_48_, with the parasitaemia of one participant not exceeding 10,000 parasites/mL until Day 10, two days later than generally occurs with 3D7-V2.

The parasite growth parameters of the 3D7-V1 in this study were similar to those obtained when the same non-linear growth model was applied to the data from the initial donor re-infected with 3D7-V1 in 1995 [19, 25]. The estimated PMR_48_ in the two subjects in this trial was 11.5 (95% CI 8.5–15.6) compared to 6.4 (95% CI 4.6–8.8) in the original volunteer inoculated in 1995. This suggests that loss of viability of the parasites after cryopreservation for over twenty years was not the reason for the slower PMR.

One possible explanation for slower than expected growth of the 3D7-V1 MCB was the lower number of infected red cells in this inoculum. Because the parasite concentrations in the individual MCBs were substantially different (< 0.01% vs 4.3% for 3D7-V1 MCB and 3D7-MBE-008, respectively), the 3D7-MBE-008 participants likely received a higher mean dose of erythrocytes infected with ring stage parasites. Alternatively, host factors may have played a factor. The participants in the 3D7-V1 study were younger than those in the MBE-008 study; however, analysis of data from previous studies has not found an association between age and PMR_48_ [25].

In addition to the slower growth rate of the 3D7-V1, another limitation for the 3D7-V1 MCB is that the original donor is Rh(D) positive, thus precluding Rh negative females from enrolling in studies using this isolate due to the risk of red cell allo-immunization. As a consequence of this and the associated slower PMR, it is likely that the 3D7-V1 MCB will be less suitable for use in further IBSM studies.

The adverse events reported for both studies are in keeping with previous IBSM studies [9–11, 28, 29]. The asymptomatic raised liver enzymes, with no associated significant rise in bilirubin, have been reported in previous IBSM studies [30, 31], sporozoite VIS [32] and in naturally occurring malaria [30, 33]. Similarly, the reduction in white cell counts, especially lymphopenia and neutropenia have previously been reported in IBSM VIS, sporozoite VIS and clinical malaria [12, 16, 34–36].

Both MCBs had similar parasite clearance profiles, with no recrudescence, further confirming that both MCBs are safe to use in malaria VIS. With the development of the biomanufactured MCB, there are now two further *P. falciparum* 3D7 MCBs that can be used in IBSM VIS. These are in addition to previously developed MCBs of non-falciparum species including *Plasmodium vivax* and *Plasmodium malariae* [11, 37, 38]. The development of these MCBs in falciparum and non-falciparum species may in future also enable IBSM VIS to be conducted in malaria endemic populations, to gain further understanding of host-immune response and to evaluate anti-malarial drug efficacy in participants who are regularly exposed to natural malaria.

A limitation of this study was that the MCBs were each evaluated in only two participants. There is a need to, therefore, be cautious in comparing the parasite growth rates of the new MCBs against the established 3D7-V2 MCB, especially as there is variability within the observed growth rates and individual growth parameters within the 3D7-V2 MCB [25]. However, when comparing the growth rates of both 3D7-MBE-008 and 3D7-V2, the individual parameters are similar (Additional file 1: Table S2).

## Conclusion

A newly developed *P. falciparum* new MCB which is safe to use in healthy, malaria-naive participants is reported. The growth characteristics of the bioreactor-expanded *P. falciparum* 3D7-MBE-008 MCB have been demonstrated and are comparable to the existing 3D7 MCB, hence this new bank is suitable for use in future studies.

## Supplementary Information

**Additional file 1: Table S1. **Schedule of events for both studies. **Table S2. **Growth parameters of the 3D7-MBE-008 pilot bank (n = 2) compared to 3D7 bank using historical data (n = 177). **Table S3. **Growth parameters of the 3D7-V1 pilot bank (n = 2) compared to 3D7-V2 bank using historical data (n = 177).** Table S4. **Overall clinical score recorded for each participant during 3D7-MBE-008 study.** Table S5. **Overall clinical score recorded for each participant during 3D7-V1 study.

## Data Availability

The datasets used and/or analysed during the current study are available from the corresponding author on reasonable request.

## Abbreviations

ALT: Alanine aminotransferase
AST: Aspartate aminotransferase
BMI: Body mass index
Co-I: Co-investigator
DBP: Diastolic blood pressure
ECG: Electrocardiogram
GMP: Good Manufacturing Procedures
HBV: Hepatitis B Virus
HCV: Hepatitis C Virus
HIV: Human Immunodeficiency Virus
HR: Heart rate
HRP2: Histidine-rich protein 2
HREC: Human research ethics committee
IBSM: Induced blood-stage malaria
LDA: Limiting dilution assay
LLN: Lower limit of normal
MCB: Malaria Cell Bank
MMV: Medicines for Malaria Venture
Pf: *Plasmodium falciparum*
*Pf* GLURP: *Pf* Glutamate-rich protein
*Pf* MSP-1/2: *P. falciparum* Merozoite surface protein 1/2
PI: Principal investigator
PMR: Parasite multiplication rate
PRR: Parasite reduction ratio
qPCR: Quantitative Polymerase Chain Reaction
RNA: Ribonucleic acid
SBP: Systolic blood pressure
spp.: Subspecies
ULN: Upper limit of normal
VIS: Volunteer infection studies
WGS: Whole genome sequencing
