# Supplementary material for: Development and evaluation of a new Plasmodium falciparum 3D7 blood stage malaria cell bank for use in malaria volunteer infection studies
Source: Malar J. 2021 Feb 16;20:93. doi: 10.1186/s12936-021-03627-z (PMC7885253; doi:10.1186/s12936-021-03627-z)
Supplement: Supplementary file 1 — Additional file 1: Table S1. Schedule of events for both studies. Table S2. Growth parameters of the 3D7-MBE-008 pilot bank (n = 2) compared to 3D7 bank using historical data (n = 177). Table S3. Growth parameters of the 3D7-V1 pilot bank (n = 2) compared to 3D7-V2 bank using historical data (n = 177). Table S4. Overall clinical score recorded for each participant during 3D7-MBE-008 study. Table S5. Overall clinical score recorded for each participant during 3D7-V1 study. [file 12936_2021_3627_MOESM1_ESM.pdf]

The development and evaluation of a novel *P. falciparum* blood stage malaria cell bank for use in malaria volunteer infection studies

Eligibility criteria for both studies

Inclusion criteria

Demography

1. Male aged 18 to 55 years inclusive who will be contactable and available for the duration of the trial and up to 2 weeks following the EOS visit.

2. Total body weight greater than or equal to 50 kg, and a body mass index (BMI) within the range of 18 to 32 kg/m^2 (inclusive). BMI is an estimate of body weight adjusted for height. It is calculated by dividing the weight in kilograms by the square of the height in metres.

Health status

3. Certified as healthy by a comprehensive clinical assessment (detailed medical history, complete physical examination and special investigations).

4. Vital signs at screening and pre-inoculation (measured after 5 minutes in the supine position):
   - Systolic blood pressure (SBP) - 90-140 mmHg,
   - Diastolic blood pressure (DBP) - 40-90 mmHg,
   - Heart rate (HR) - 40-100 bpm.

5. At screening and pre-inoculation: QTcF ≤450 ms, QTcB ≤450 ms (male participants); PR interval ≤210 ms.

6. Male participants must agree to use a double method of contraception including condom plus diaphragm, or condom plus intrauterine device, or condom plus stable oral/transdermal/injectable hormonal contraceptive by the female partner, from the time of informed consent until the end of artemether-lumefantrine treatment. Abstinent male participants must agree to start a double method if they begin sexual relationships during the study, and until the end of artemether-lumefantrine treatment. Male participants with female partners that are surgically sterile, or male participants who have undergone sterilisation and have had testing to confirm the success of the sterilisation, may also be included.

Regulations

7. Completion of the written informed consent process prior to undertaking any study-related procedure.
8. Must be willing and able to communicate and participate in the whole study.

Exclusion criteria

Participants fulfilling any of the following criteria are not eligible for inclusion in this study:

Medical history and clinical status

1. Participant with Rhesus (D) Negative blood group (For 3D7-V1 only).
2. Participant lives alone (at any stage from inoculation day until the end of the artemether-lumefantrine treatment).

3. Any history of malaria or participation in a previous malaria challenge study or malaria vaccine trial.

4. Must not have travelled to or lived (>2 weeks) in a malaria-endemic region during the past 12 months or planned travel to a malaria-endemic region during the course of the study. Must not have lived for >1 year in a malaria-endemic region in the past 10 years. Must not have ever lived in a malaria-endemic region for more than 10 years inclusive. For endemic regions see https://map.ox.ac.uk/country-profiles/#!/. Bali is not considered a malaria-endemic region.

5. Has evidence of increased cardiovascular disease risk (defined as >10%, 5-year risk for those greater than 35 years of age, as determined by the Australian Absolute Cardiovascular Disease Risk Calculator (http://www.cvdcheck.org.au/)). Risk factors include sex, age, systolic blood pressure (mm/Hg), smoking status, total and HDL cholesterol (mmol/L), and reported diabetes status.

6. History of splenectomy.

7. Participant unwilling to defer blood donations to the Blood Service for at least 6 months after the End of Study visit.

8. Participant who has ever received a blood transfusion.

9. Any recent (<6 weeks) or current systemic therapy with an antibiotic or drug with potential antimalarial activity (e.g. chloroquine, piperaquine phosphate, benzodiazepine, flunarizine, fluoxetine, tetracycline, azithromycin, clindamycin, doxycycline etc.).

10. Known hypersensitivity to artesunate or any of its excipients, artemether or other artemisinin derivatives, proguanil/atovaquone, primaquine, or 4-aminoquinolines.

11. Haematology, clinical chemistry or urinalysis results at screening or at the Day -3 to -1 eligibility visit that are outside of Sponsor-approved clinically acceptable laboratory ranges or are considered clinically significant by the Sub-Investigator.
12. Participation in any investigational product study within the 12 weeks preceding inoculation with the malaria challenge agent.
13. Symptomatic postural hypotension at screening (confirmed on two consecutive readings), irrespective of the decrease in blood pressure, or asymptomatic postural hypotension defined as a decrease in systolic blood pressure ≥20 mmHg within 2-3 minutes when changing from supine to standing position.
14. History or presence of diagnosed (by an allergist/immunologist) or treated (by a physician) food or known drug allergies (including but not limited to allergy to any of the antimalarial rescue medications), or history of anaphylaxis or other severe allergic reactions. Participants with seasonal allergies/hay fever or allergy to animals or house dust mite that are untreated and asymptomatic at the time of dosing can be enrolled in the study.
15. History of convulsion (including intravenous drug or vaccine-induced episodes). A medical history of a single febrile convulsion during childhood is not an exclusion criterion.
16. Presence of current or suspected serious chronic diseases such as cardiac or autoimmune disease (HIV or other immuno-deficiencies), insulin-dependent and non-insulin dependent diabetes (excluding glucose intolerance if exclusion criterion 5 is met), progressive neurological disease, severe malnutrition, acute or progressive hepatic disease, acute or progressive renal disease, porphyria, psoriasis, rheumatoid arthritis,

asthma (excluding childhood asthma, or mild asthma with preventative asthma medication required less than monthly), epilepsy, or obsessive-compulsive disorder.

17. History of malignancy of any organ system (other than localised basal cell carcinoma of the skin or in situ cervical cancer), treated or untreated, within 5 years of screening, regardless of whether there is evidence of local recurrence or metastases.
18. Participants with history of schizophrenia, bi-polar disease, psychoses, disorders requiring lithium, attempted or planned suicide, or any other severe (disabling) chronic psychiatric diagnosis.
19. Participants who have received psychiatric medications within 1 year prior to enrolment, or who have been hospitalised within 5 years prior to enrolment for either a psychiatric illness or due to danger to self or others.
20. History of more than one previous episode of major depression, any previous single episode of major depression lasting for or requiring treatment for more than 6 months, or any episode of major depression during the 5 years preceding screening.

    The Beck Depression Inventory will be used as an objective tool for the assessment of depression at screening. In addition to the conditions listed above, participants with a score of 20 or more on the Beck Depression Inventory and/or a response of 1, 2 or 3 for item 9 of this inventory (related to suicidal ideation) will not be eligible for participation. These participants will be referred to a general practitioner or medical specialist as appropriate. Participants with a Beck score of 17 to 19 may be enrolled at the discretion of the Principal investigator (PI) or Co-investigator (Co-I) if they do not have a history of the psychiatric conditions mentioned in this criterion and their mental state is not

considered to pose additional risk to the health of the participant or to the execution of the study and interpretation of the data gathered.

21. History of recurrent headache (e.g. tension-type, cluster or migraine) with a frequency of ≥2 episodes per month on average and severe enough to require medical therapy, during the 2 years preceding screening.
22. Presence of clinically significant infectious disease or fever (e.g. sublingual temperature ≥38.5°C) within the 5 days prior to inoculation with the malaria challenge agent.
23. Evidence of acute illness within the 4 weeks prior to screening that the Sub-Investigator deems may compromise participant safety.
24. Significant inter-current disease of any type, in particular liver, renal, cardiac, pulmonary, neurologic, rheumatologic, or autoimmune disease by history, physical examination, and/or laboratory studies including urinalysis.
25. Participant has a clinically significant disease or any condition or disease that might affect drug absorption, distribution or excretion (e.g. gastrectomy, diarrhoea).
26. Blood donation of any volume within 1 month before inclusion, or participation in any research study involving blood sampling (more than 450 mL/unit of blood), or blood donation to Australian Red Cross Blood Service (Blood Service) or other blood bank during the 8 weeks prior to the reference drug dose in the study.
27. Medical requirement for intravenous immunoglobulin or blood transfusions.
28. Any vaccination within the last 28 days.
29. Any corticosteroids, anti-inflammatory drugs (excluding commonly used over-the-counter anti-inflammatory drugs such as ibuprofen, acetylsalicylic acid, diclofenac),

immunomodulators or anticoagulants within the past 3 months. Any participant currently receiving or having previously received immunosuppressive therapy (including systemic steroids, adrenocorticotrophic hormone or inhaled steroids) at a dose or duration potentially associated with hypothalamic-pituitary-adrenal axis suppression within the past year.

30. Use of prescription drugs or non-prescription drugs or herbal supplements (such as St John's Wort), within 14 days or 5 half-lives (whichever is longer) prior to or inoculation with the malaria challenge agent. As an exception, ibuprofen (preferred) may be used at doses of up to 1.2 g/24 hours or paracetamol at doses of up to 4 g/24 hours after discussion with the PI or Co-I. Limited use of other non-prescription medications or dietary supplements, not believed to affect participant safety or the overall results of the study, may be permitted on a case-by-case basis following approval by the PI or Co-I. Participants are requested to refrain from taking non-approved concomitant medications from recruitment until the conclusion of the study.

General conditions

31. Any participant who, in the judgment of the PI or Co-I, is likely to be non-compliant during the study, or is unable to cooperate because of a language problem or poor mental development.

32. Any participant in the exclusion period of a previous study according to applicable regulations.

33. Any participant who is the PI, Co-I or any Sub-Investigator, research assistant, pharmacist, study coordinator, or other staff thereof, directly involved in conducting the study.

34. Any participant without a good peripheral venous access.

Biological status

35. Positive result on any of the following tests: hepatitis B surface antigen (HBs Ag), anti-hepatitis B core antibodies (anti-HBc Ab), anti-hepatitis C virus (anti-HCV) antibodies, anti-human immunodeficiency virus 1 and 2 antibodies (anti-HIV1 and anti-HIV2 Ab).

36. Positive urine drug test. Any drug listed in Section 8.2 in the urine drug screen unless there is an explanation acceptable to the Sub-Investigator (e.g., the participant has stated in advance that they consumed a prescription or over-the-counter product which contained the detected drug) and/or the participant has a negative urine drug screen on retest by the pathology laboratory. Any participant testing positive for acetaminophen (paracetamol) at screening and/or inoculation day may still be eligible for study participation, at the PI or Co-I's discretion.

37. Positive alcohol breath test.

Specific to the study

38. Cardiac/QT risk:
    - Family history of sudden death or of congenital prolongation of the QTc interval or known congenital prolongation of the QTc interval or any clinical condition known to prolong the QTc interval.
    - History of symptomatic cardiac arrhythmias or with clinically relevant bradycardia.

- Electrolyte disturbances, particularly hypokalaemia, hypocalcaemia, or hypomagnesaemia.
- ECG abnormalities in the standard 12-lead ECG (at screening and prior to inoculation with the malaria challenge agent) which in the opinion of the Sub-Investigator is clinically relevant or will interfere with the ECG analyses.

39. History or presence of alcohol abuse (alcohol consumption more than 40 g/4 units/4 standard drinks per day), or drug habituation, or any prior intravenous usage of an illicit substance.
40. Tobacco use of more than 5 cigarettes or equivalent per day, and unable to stop smoking for the duration of the clinical unit confinement.
41. Ingestion of any poppy seeds within the 24 hours prior to screening (participants will be advised by phone not to consume any poppy seeds in this time period).
42. Excessive consumption of beverages or food containing xanthine bases including Red Bull, chocolate, coffee etc. (more than 400 mg caffeine per day, equivalent to more than 4 cups of coffee per day).
43. Unwillingness to abstain from consumption of quinine containing foods/beverages such as tonic water and lemon bitter from inoculation day until the end of the artemether-lumefantrine treatment.
44. Unwillingness to abstain from consumption of grapefruit or Seville oranges from inoculation day until the end of the artemether-lumefantrine treatment.

Table S1. Schedule of events for both studies

**Procedures**	**Screening**	**Safety Visit required)**[a]	**Challenge Inoculation**	**Malaria Monitoring**		**Drug Treatment (Confinement)**			**Post-treatment Follow-up (outpatient)**			**Safety Assessment**		**EOS**
Day	**-Day 28 to Day -1**	**Day -3 to Day -1**	**Day 0**	**Phone Contact Day 1 – Day 3**	**Day 4 until artemether lumefantrine treatment**[b]	**First dose (0hrs)**	**Second dose (12hrs)**	**Third dose (24hrs)**	**36hrs**	**48hrs**	**72hrs**	**Day 30±2**	**Day 60±2**	**Day 90±2**
Eligibility Assessments														
Informed consent	X													
Beck Depression Inventory	X													
Demography	X													
Medical history, inc/exc. criteria, & prior medications	X		X											
Drug & alcohol screen	X		X											
Body weight	X										X			
Height	X													
Serology	X													X
RBC alloantibody	X													X
Coagulation profile	X													
G6PD testing	X													
Safety Assessments														
Complete Physical exam	X										X			
Abbreviated physical exam			X			X								
Symptom-directed physical exam					Throughout the period when clinically indicated									
ECGs	X		X			X		X			X			

**Procedures**	**Screening**	**Safety Visit required)[a]**	**Challenge Inoculation**	**Malaria Monitoring**		**Drug Treatment (Confinement)**			**Post-treatment Follow-up (outpatient)**			**Safety Assessment**		**EOS**
Vital signs	X		X		Throughout the period									
Haematology & Biochemistry	X	X				X		X			X			
Urinalysis	X	X				X					X			
Safety serum storage			X								X			
Diary card			X	X	X				X	X	X			
Adverse Events			Throughout the period									X	X	X
Malaria Clinical Score[d]			X		Throughout the period									
Phone Call												X	X	
Other														
Blood Stage Challenge Agent			X											
Artemether-lumefantrine Treatment						X	X	X	X	X				
Artemether-lumefantrine Treatment phone call/text[e]										X				
Malaria 18S qPCR blood sampling[f]			X		X	X	X	X	X	X	X			

Table S2. Growth parameters of the 3D7-MBE-008 pilot bank (n=2) compared to 3D7 bank using historical data (n=177)

Parameter	3D7-MBE-008 Bank (n=2) Estimate (SE)	3D7-V2 Bank (n=177) Estimate (SE)	Q_B	p value
Growth rate (log₁₀ parasites/day)	0.770 (0.073)	0.752 (0.012)	0.058	0.81
Amplitude ^ **(log₁₀ parasites/day)**	0.804 (0.144)	0.629 (0.018)	1.44	0.23
Life- cycle (days)	1.615 (0.046)	1.615 (0.009)	0.00	1.00

^absolute value of amplitude

Q_B, test statistic from the omnibus test for heterogeneity

SE, Standard Error

Non-linear mixed effect model parameter estimates from the 3D7-MBE-008 bank and from historical 3D7 bank are given in Table S2, along with the results from the tests of heterogeneity of the parameter estimates. The results from the heterogeneity test suggests that there is no evidence of a difference in any of the growth parameters between the banks.

Table S3. Growth parameters of the 3D7-V1 pilot bank (n=2) compared to 3D7-V2 bank using historical data (n=177)

**Parameter**	**3D7-V1 Bank (n=2) Estimate (SE)**	**3D7-V2 Bank (n=177) Estimate (SE)**	$\boldsymbol{Q_B}$	**p value**
Growth rate (log$_{10}$ parasites/day)	0.530 (0.035)	0.752 (0.012)	35.3	<0.001
***Amplitude ^* (log$_{10}$ parasites/day)**	0.521 (0.118)	0.629 (0.018)	0.8	0.37
Life- cycle (days)	1.522 (0.033)	1.615 (0.009)	7.5	0.006

^absolute value of amplitude

$\boldsymbol{Q_B}$, test statistic from the omnibus test for heterogeneity

SE, Standard Error

Non-linear mixed effect model parameter estimates from the 3D7-V1 bank and from historical 3D7-V2 bank are given in Table S3, along with the results from the tests of heterogeneity of the parameter estimates. Evidence of a difference in the growth rate was noted between the banks.

Table S4. Overall clinical score recorded for each participant during 3D7-MBE-008 study

Days post-inoculation	Overall clinical score	
	R101	R201
0	0	0
3	-	1
4AM	0	2
4PM	0	1
5AM	0	0
5PM	0	0
6AM	0	0
6PM	1	1
7AM	1	0
7PM	3	1
8 Pre-dose	1	1
Adm +8h	9	1
Adm +12h	4	1
Adm + 24h	3	1
Adm + 36h	2	2
Adm + 48h	1	-
Adm + 72h	0	-

Table S5. Overall clinical score recorded for each participant during 3D7-V1 study

Days post-inoculation	Overall clinical score	
	R101	R201
0	0	0
3	0	0
4AM	0	0
4PM	0	-
5AM	0	0
5PM	1	-
6AM	0	0
6PM	0	0
7AM	0	0
7PM	0	0
8AM	0	0
8PM	0	0
9AM	0	0
9PM	1	0
10AM	0	0
10PM	0	2
11AM	0	3
11PM	0	0
12	1	-
Adm +8h	1	1
Adm +12h	1	4
Adm + 24h	0	1
Adm + 36h	3	8
Adm + 48h	2	-
Adm + 72h	1	-
